# Effect of Microstructure and Hardness on Cavitation Erosion and Dry Sliding Wear of HVOF Deposited CoNiCrAlY, NiCoCrAlY and NiCrMoNbTa Coatings

**DOI:** 10.3390/ma15010093

**Published:** 2021-12-23

**Authors:** Mirosław Szala, Mariusz Walczak, Aleksander Świetlicki

**Affiliations:** Department of Materials Engineering, Faculty of Mechanical Engineering, Lublin University of Technology, 20-618 Lublin, Poland; m.walczak@pollub.pl

**Keywords:** cavitation corrosion, wear, surface engineering, roughness, nickel, cobalt, tribology, hardness, erosion rate, failure analysis, MCrAlY

## Abstract

Metallic coatings based on cobalt and nickel are promising for elongating the life span of machine components operated in harsh environments. However, reports regarding the ambient temperature tribological performance and cavitation erosion resistance of popular MCrAlY (where M = Co, Ni or Co/Ni) and NiCrMoNbTa coatings are scant. This study comparatively investigates the effects of microstructure and hardness of HVOF deposited CoNiCrAlY, NiCoCrAlY and NiCrMoNbTa coatings on tribological and cavitation erosion performance. The cavitation erosion test was conducted using the vibratory method following the ASTM G32 standard. The tribological examination was done using a ball-on-disc tribometer. Analysis of the chemical composition, microstructure, phase composition and hardness reveal the dry sliding wear and cavitation erosion mechanisms. Coatings present increasing resistance to both sliding wear and cavitation erosion in the following order: NiCoCrAlY < CoNiCrAlY < NiCrMoNbTa. The tribological behaviour of coatings relies on abrasive grooving and oxidation of the wear products. In the case of NiCrMoNbTa coatings, abrasion is followed by the severe adhesive smearing of oxidised wear products which end in the lowest coefficient of friction and wear rate. Cavitation erosion is initiated at microstructure discontinuities and ends with severe surface pitting. CoNiCrAlY and NiCoCrAlY coatings present semi brittle behavior, whereas NiCrMoNbTa presents ductile mode and lesser surface pitting, which improves its anti-cavitation performance. The differences in microstructure of investigated coatings affect the wear and cavitation erosion performance more than the hardness itself.

## 1. Introduction

Thermal spraying processes are commonly used methods for metallic materials restoration and modification of surface layer properties that are applied in many industries. The process is used in the aerospace industry, automotive engineering, and maritime sectors [1,2,3]. One of the most popular thermal spraying methods is the HVOF (high velocity oxygen-fuel). The HVOF method is most often used because the process is characterized by a high particle velocity, relatively low temperature and a short time of particle exposure in the stream, which results in a low content of oxides and minimal porosity [4,5]. In comparison to other thermal spraying techniques such as arc, flame spraying, or APS coatings deposited using the HVOF, they are usually characterized by high adhesion to the substrate and low porosity [6,7,8,9]. This technology allows for the extension of the service life of materials, the improving of the mechanical properties, and the increase in the operational performance of the engineering devices. These features determine the economic benefits of HVOF-deposited coatings. Systematic broadening of the coatings industrial applications followed by the diversity of the feedstock materials makes the thermally sprayed coatings a crucial subject of recent scientific papers.

A broad range of materials is deposited via the HVOF method, such as metals, ceramics and composites. Overall, the main application of thermally sprayed coatings is protection against different types of wear [10,11,12,13], and more sophisticated implementation involves the prevention from cavitation erosion [14,15,16]. Besides, industrial applications combine these two deterioration processes, such as pressure values, diesel liners or specialised chemical equipment. As an example, MCrAlY coatings (where M = Co, Ni or Co/Ni) are widely used in the aviation industry, as MCrAlY is used as the bond coat of TBCs or as a standalone protective coating to increase resistance to high temperatures and high pressure occurring in gas turbines [17,18]. Therefore, the wear resistance of MCrAlY and NiCoMo coatings is most often analysed in publications in terms of high-temperature environments [19,20,21,22]. Few studies describe the behaviour of these coatings at room temperature, and to the best of the authors′ knowledge, there is no comparative analysis for the MCrAlY and NiCrMo coatings in terms of dry sliding wear and cavitation erosion behavior.

Similarly to tribological deterioration, cavitation erosion (CE) can harm the service life of machinery and equipment [23,24,25,26]. Therefore, HVOF coatings constituted of nickel and/or cobalt-based feedstock powders are often used as a protective layer [16,27,28,29]. However, in terms of cavitation erosion, it is believed that cobalt-based materials present one of the highest cavitation erosion resistances [28,30,31]. On the other hand, the nickel-based powders also give interesting anti-cavitation erosion results [32,33,34]. Still, the effect of the metallic matrix type, influencing microstructure and other properties, is not clearly stated, especially in the case of HVOF Ni/Co-based coatings, including MCrAlY and NiCrMo. Besides, nickel-based coatings present desirable properties in anti-wear and cavitation, preventing applications due to the availability of various deposition methods, high adhesion of the coating to the substrate, and resistance to corrosion required in the wet environment. Furthermore, plastic deformation of nickel-based coatings may also reduce the rate of CE damage of the material by absorbing the energy generated by cavitation pressure waves and micro-jets [35,36]. Therefore, it seems interesting to investigate the CE resistance of HVOF coatings containing nickel and clarify the effect of coatings properties on the CE behavior.

Furthermore, depositing a protective MCrAlY and NiCrMo coating on a base metal substrate is usually studied concerning their typical high-temperature operation conditions [36,37], while their sliding behaviour at ambient temperature and anti-cavitation performance are not thoroughly investigated by the literature of the object [38]. Nevertheless, a well-known fact is that cobalt-based HVOF metallic [28,39,40] and Co-WC, CoCr-WC [41,42,43] cermet coatings present superior tribological performance and resistance to cavitation erosion. The anti-cavitation properties of Ni/Co-based HVOF deposits are not completely discussed. Mainly, limited studies describe the research on CE resistance for MCrAlY, NiCoMo deposits. Therefore, this study comparatively analysed the coatings microstructure and properties with a resistance to cavitation erosion and dry sliding wear at ambient temperature. The results give useful remarks for broadening the knowledge regarding dry sliding wear and cavitation erosion failure mechanisms of MCrAlY and NiCrMo coatings.

This work aimed to investigate the effect of microstructure and hardness of HVOF sprayed metallic coatings on their tribological and cavitation erosion performance. Moreover, sliding wear and cavitation erosion mechanisms were studied. The research analysed commercial HVOF coatings deposited from popular nickel-based feedstock powders: CoNiCrAlY, NiCoCrAlY and NiCrMoNbTa.

## 2. Materials and Methods

### 2.1. Materials

In this article, three coatings thermally sprayed via the HVOF (High Velocity Oxygen Fuel) method using popular commercial powders were examined to state the effect of nickel content on tribological and cavitation erosion (CE) behaviour. (Ni,Co)CrAlY and NiCrAlNbTa feedstock powders were sprayed on 2 mm thick Inconel 617 sandblasted substrates and thickness of coatings yielding 100 ± 25 µm. The nominal chemical composition of substrate and feedstock powders are given in Table 1 and Table 2, respectively.

The CoNiCrAlY coating was fabricated using cobalt-based powder Amdry 9954 manufactured by Oerlikon Metco (Pfäffikon, Switzerland), which is intended to protect surfaces against oxidation and corrosion at high temperatures above 850 °C. The second coating of NiCoCrAlY nickel-based powder is made of a powder called Ni-191-4 (PRAXAIR), which is also designed to produce high-temperature layers, protecting against oxidation and high-temperature corrosion. The last tested NiCrMoNbTa coating was sprayed using spheroidal powder with the trade name Diamalloy 1005 (Oerlikon Metco), which is dedicated to surface regeneration, protection of the surface against oxidation and corrosion at high temperatures above 982 °C. MCrAlY and NiCrMoNbTa coatings are usually studied concerning their typical high-temperature operating conditions. The other practical applications are not identified. Thus, this paper comparatively analyses their tribological and anti-cavitation performance.

### 2.2. Research Methodology

The coatings were investigated using scanning electron microscopy (SEM), X-ray phase analysis (XRD) and profilometer measurements. The surface morphology of the as-sprayed coatings and cross-section microstructures of the coatings were examined using the SEM-EDS method. The phase composition of the coatings was investigated by the X-ray diffraction method. The phase composition studies were carried out according to the procedure described in a previous paper [38]. The surface roughness Ra parameter was determined using the surface profiler (Surtronic S-128, Taylor-Hobson, Leicester, UK) according to the ISO 4287 standard. Finally, the hardness of the tested coatings was measured following the PN-EN ISO 6507-1 standard. The tests were carried out on polished transverse specimens. Investigations were done with the use of the load of 0.9807 N and the dwelling time of 10 s. At the latest, ten indentations were made to obtain statistical accuracy.

**Figure 1:**
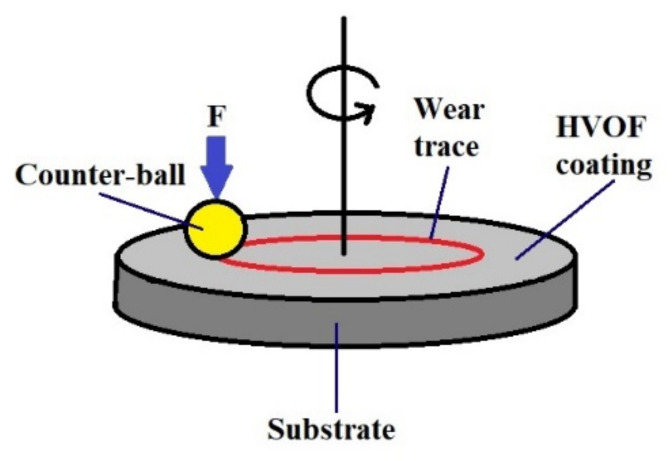
Scheme of ball-on-disc wear testing.

In order to compare the wear resistance of as-sprayed HVOF coatings with other engineering materials tested by our group [44,45,46] the ball-on-disc method was employed. Wear tests were carried out on a CS-Instruments ball tribotester. The idea of the wear test is presented in Figure 1. As a counter-sample (ball), six mm diameter balls made of tungsten carbide-cobalt (WC–Co) cemented carbide (supplied by CSM Instruments, Needham Heights, MA, USA) were used. The tests were carried out under an applied load of F = 10 N. The tests were carried out with a linear speed of 0.05 m/s for a radius of 3 mm and a sliding distance of 200 m. Test conditions prevent local abrasion of coatings to the substrate. The friction coefficient was also determined. Additionally, the wear factor K was determined using the procedure and the Equation (1) described in a previous paper [44].

(1) $$K=\frac{volume\;loss\;\left[mm^{3}\right]}{load\;\left[N\right]\cdot sliding\;distance\;\left[m\right]}$$

Additionally, this research aimed to test the wear resistance of the as-sprayed coatings, i.e., investigated coatings differ in surface roughness. Therefore sliding wear resistance (estimated by wear factor) was validated using the weighting method. HVOF coatings mass loss was measured with an accuracy of 0.1 mg. Finally, the sliding wear mechanism was comparatively investigated using the SEM-EDS method.

**Figure 2:**
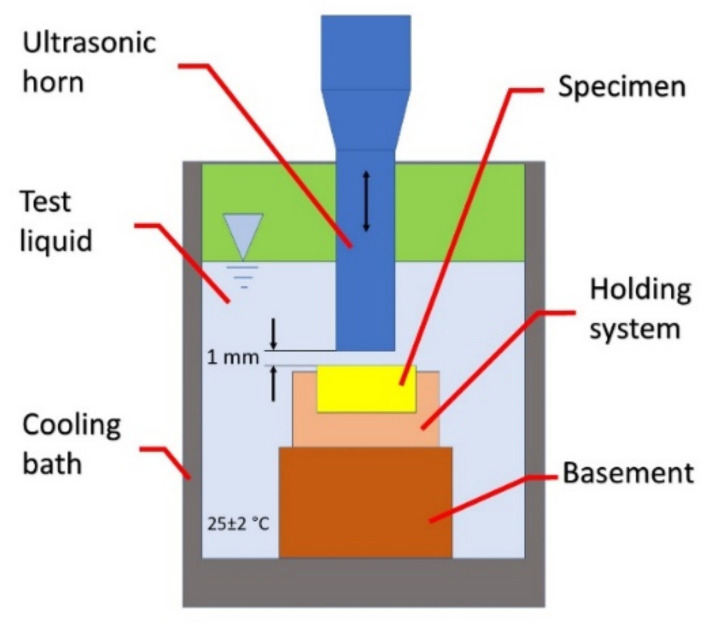
Schematic representation of the ultrasonic vibratory system used for cavitation.

The cavitation erosion resistance tests were carried out in accordance with the ASTM G32 standard [47]. The sonotrode tip distance from the sample was 1 mm ± 0.05 mm, the medium in which cavitation was induced was distilled water, see Figure 2. The tip working area equals 1.92 cm^2^. The analysis of the resistance to cavitation erosion consisted of systematic measurements of the weight loss of the tested samples with an accuracy of 0.01 mg. The cavitation testing conditions conform to the previous research conditions done for APS [45], HVOF [48], cold spray [49] and electrostatic [50] deposits investigated by our group. In the current study, the total exposure time lasted 3 h. The study cavitation erosion mechanisms, surfaces of test samples were finished by grinding with emery papers # 240, # 320 and # 600 to achieve the surface roughness of Ra < 5.28 µm, Rt < 38.3 µm and Rz < 28.5 µm. At stated time intervals of the cavitation erosion testing, the eroded surfaces roughness measurements and SEM microscopic analysis were carried out.

## 3. Results and Discussion

### 3.1. Microstructure and Coatings Properties

**Figure 3:**
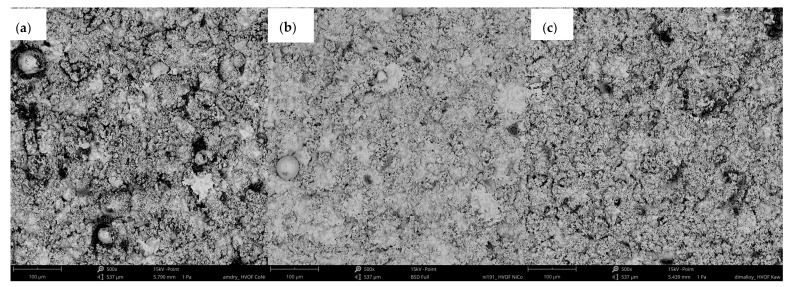
Surface morphology of as-sprayed coatings: (**a**) CoNiCrAlY; (**b**) NiCoCrAlY and (**c**) NiCrMoNbTa.

**Figure 4:**
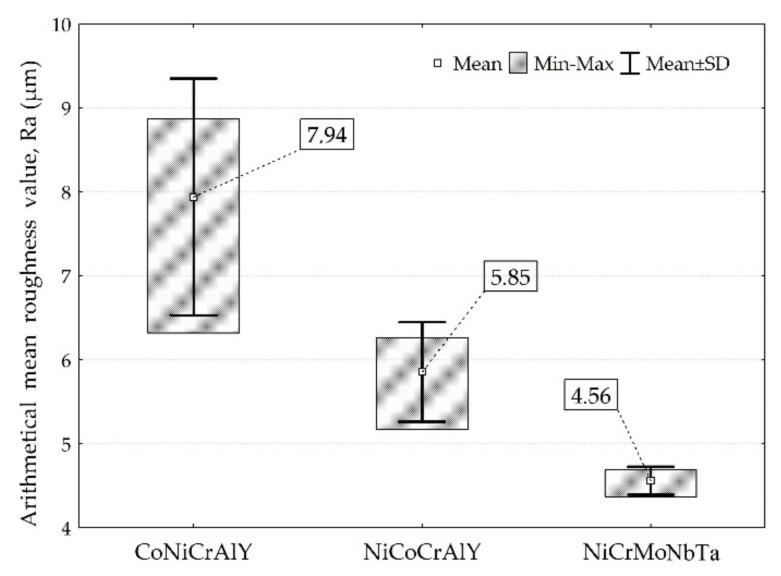
Comparison of the roughness of as-sprayed coatings.

The tests carried out showed the presence of a microstructure characteristic for thermally sprayed coatings. The as-sprayed surfaces are characterised by the presence of splats, unmelted particles and oxides (Figure 3). Much more uniform surface roughness was identified for NiCrMoNbTa than for MCrAlY’s (Figure 4). Besides, the mean value of the Ra roughness parameter was the highest for the CoNiCrAlY coating (7.94 µm) and presented the widest spread of results. The obtained value compares to the mean roughness of the same type of HVOF coating (Ra = 7.41 μm) reported by Rajasekaran et al. [51]. As-sprayed NiCoCrAlY presents surface roughness even lower than those reported by the literature for the same type of MCrAlY [52]. The smoothest surface roughness of the Ra (4.56 µm) was obtained for NiCrMoNbTa. Thus, the Ra roughness ranges between values reported by Oladijo et al. [53] and Al-Fadhli et al. [54] reported for HVOF sprayed Inconel 625 coatings (similar chemical composition to NiCrMoNbTa). According to the literature, the roughness of MCrAlY bond coat affects adhesion between the metallic bond coat and the ceramic topcoat and is an important factor for the extended TBC life [52,55]. Whereas one of the typical applications of NiCrMoNbTa is surface regeneration. Thus, the low coating roughness of NiCrMoNbTa is optimal for the restoration and repair technology of superalloy components.

**Figure 5:**
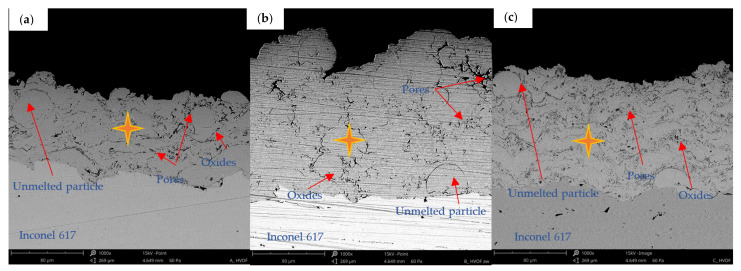
Coatings cross sections: (**a**) CoNiCrAlY, (**b**) NiCoCrAlY, (**c**) NiCrMoNbTa, SEM-EDS (spot analysis are given in Table 3).

**Figure 6:**
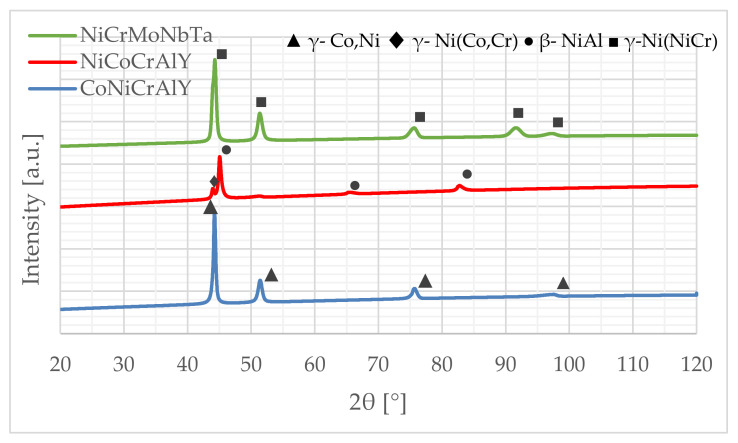
Comparison of phase composition of coatings, XRD.

**Figure 7:**
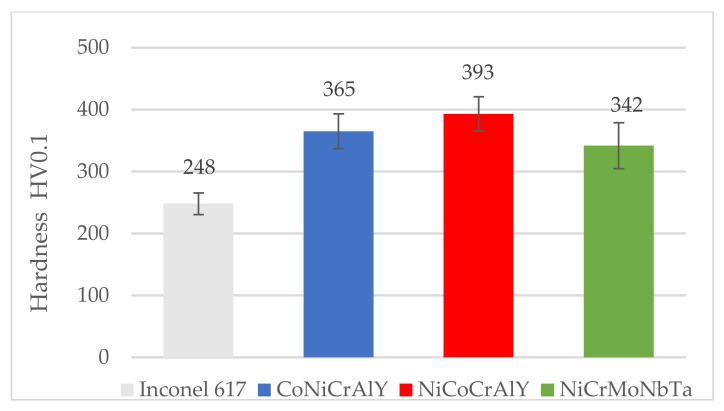
Hardness variability of coatings and the substrate.

The cross-section microstructures (Figure 5) show a sufficient interlocking between the applied coating and the substrate. The coatings have a lamellar structure, porosity, and oxides at the clear boundaries between the lamellas, and incompletely melted powder particles are visible. In the case of the NiCoCrAlY coating (Figure 5a), a much more dense structure was observed than in the case of MCrAlY coatings. The SEM-EDS investigations (Figure 5 and Table 3) reveal the percentage content of the main elements in the range of the nominal feedstock powders (Table 2). Due to the low nominal content and EDS method limitations, the yttrium was not detected, and the percentage content of other chemical elements varied. Both nickel and cobalt constitute the coatings metallic matrix and are crucial for phase composition and overall properties and anti-wear performance. Thus, the X-ray phase composition was investigated, see Figure 6. In the case of coating CoNiCrAlY the γ-Co,Ni phase with fcc structure was identified. On the other hand, the literature of the subject [56,57,58,59] suggests the presence of an intermetallic phase β-(Co,Ni)Al with bcc structure, although this phase wasn’t confirmed in CoNiCrAlY sample. The second HVOF coating NiCoCrAlY has a two-phase composition consisting of γ-Ni(Co,Cr) and β-NiAl, which follows findings obtained for MCrAlY coating deposited by HVOF [60,61]. The third sample, NiCrMoNbTa presents a single-phase structure based on γ-Ni(NiCr) which corresponds to the results obtained for HVOF fabricated NiCrMoNb-matrix composites [12,62]. The coatings’ microstructure and phase composition differs and, of course, influences coatings hardness, see Figure 7. Coatings hardness exceeds Inconel 617 substrate, namely 248.1 HV. The highest average hardness was noted for the NiCoCrAlY coating, about 393 HV. The lowest hardness was found for NiMoCrNbTa and it was 342 HV. In sum, the hardness of CoNiCrAlY exceeds those reported for NiCoCrAlY, which agrees with the trends given in the literature [21]. On the other hand, even though the mean hardness differs, it should be noted that analysis of hardness variability confirms overlapping of standard deviations, as shown in Figure 7. On the whole, the coatings nominal chemical composition affects morphology, phase composition, and hardness which are essential factors to clarify tribological and cavitation erosion performance.

### 3.2. Tribological Testing

**Figure 8:**
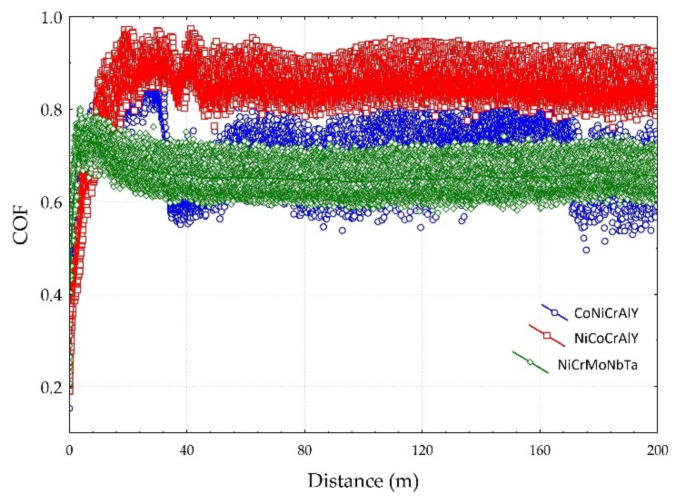
Coefficient of friction (COF) of the tested coatings.

**Figure 9:**
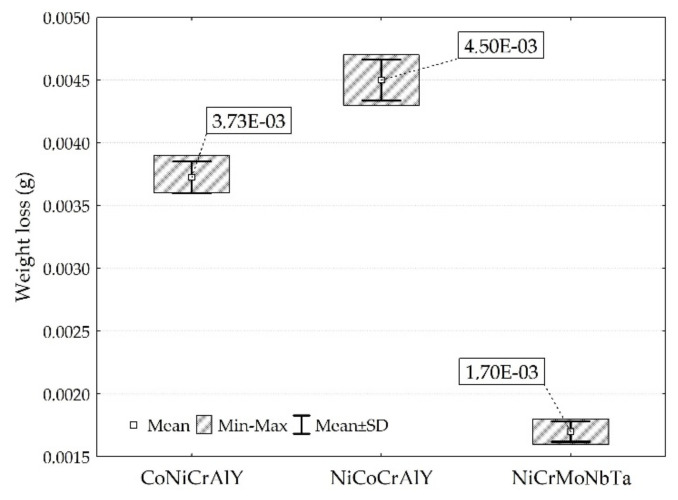
Mass losses of tested coatings.

**Figure 10:**
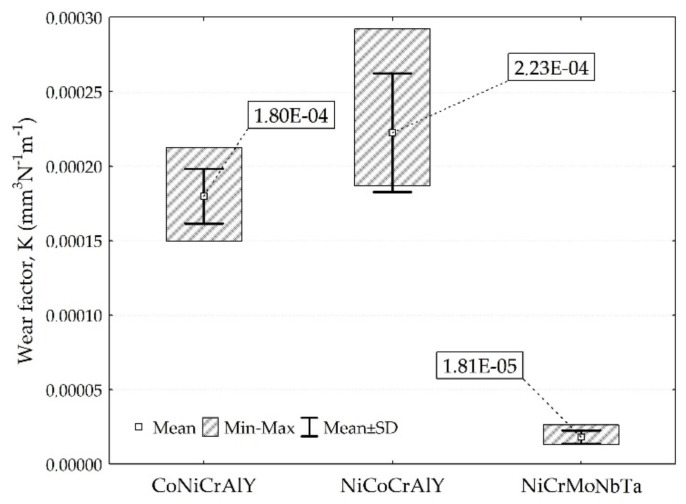
Sliding wear result of investigated coatings.

The coefficient of friction (COF) was determined for the tested coatings. The results are presented in Figure 8, while mass loss and wear factor K are shown in Figure 9 and Figure 10, respectively. The tribological tests were conducted for as-sprayed surfaces. Therefore, the accuracy of tribological results was positively validated by comparing mass losses (evaluated using the weighting method) with the volume losses (wear factors), see Figure 9 and Figure 10. In other words, utilised test conditions allow obtaining reliable tribological results for the as-sprayed coatings. Nonetheless, the investigated HVOF coatings present inferior wear behaviour compared to other MCrAlY, and NiCrMo mostly tested at elevated temperatures [13,22,63], as well as to ceramic [10,45], metallic [44,46] materials tested at the same load at ambient temperature.

**Figure 11:**
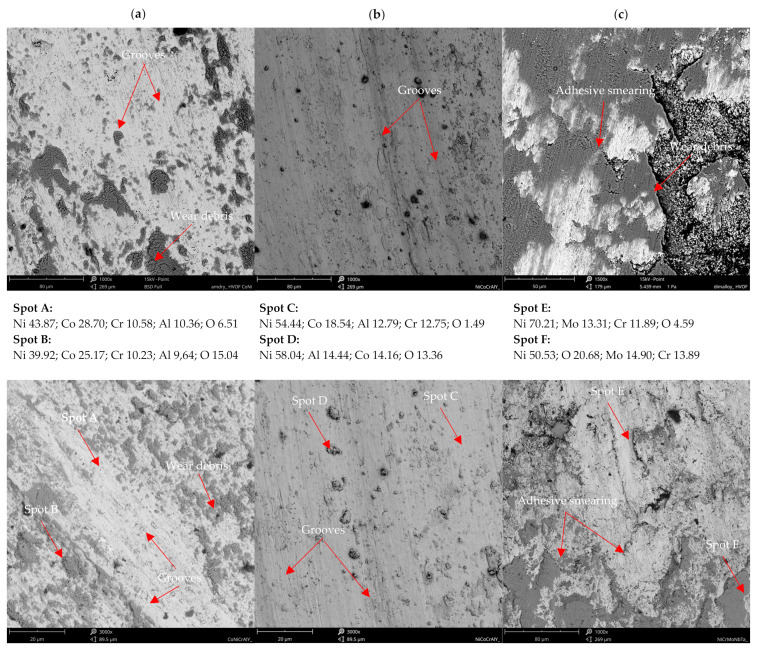
Wear traces and spot chemical analysis: (**a**) CoNiCrAlY, (**b**) NiCoCrAlY, (**c**) NiCrMoNbTa wear trace, SEM-EDS in wt.%.

The influence of mean hardness on the wear resistance of the coatings was stated. NiCoCrAlY coating with the highest mean hardness 393 HV0.1 showed the worst resistance to wear. Moreover, the coating with the lowest mean hardness 342 HV0.1 showed the highest resistance to wear. It contradicts the literature, which reports that higher hardness increases material wear resistance [64]. It should be noted that differences were identified for the mean hardness and the spread of hardness results overlaps. Therefore, it can be concluded that the microstructure of investigated coatings affects the wear performance more than the hardness itself. Even though the NiCoCrAlY coating has the highest mean hardness, the presence of the second phase could be responsible for its poor tribological performance. Besides, the NiCrMoNbTa coating has the lowest COF and material loss. At the same time, the NiCoCrAlY coating had the highest COF and K factor, which can be explained by the presence of the second phase β-NiAl. Other coatings present a single-phase structure. The wear factor of NiCrMoNbTa was almost 13 times lower than the one obtained for coating NiCoCrAlY. This can be explained by the microstructure of the coating, which affects the wear mechanisms. The NiCrMoNbTa coating is much more affected by the adhesive smearing of wear debris than the MCrAlY coatings. In the case of all samples, the abrasive wear grooving and the tribochemical wear, namely oxidation of wear products, were confirmed by the SEM-EDS investigations, as shown in Figure 11. In the case of sliding testing of as-sprayed HVOF coatings, the hard WC counter-ball smashes the rough surface, and the produced wear debris acts as a third body, which is confirmed by other researchers [65]. Then the sliding wear mechanism changes to abrasion affected by grooving. Moreover, the adhesive transfer of smeared wear products is visible in CoNiCrAlY and NiCrMoNbTa coatings, shown in Figure 11a,b, which affects the decrease of wear factor and COF in comparison to NiCoCrAlY. Finally, their wear mechanism is supported by fatigue which is observed especially for wear debris. Fatigue took place as a result of repeated movement of the counter-sample on the surface of the coating and initiated microcracks, which proceeds and finally causes the material detachment and transfer through the wear tack. In the case of the NiCrMoNbTa coating shown in Figure 11c, the abrasive grooving followed by adhesive smearing of oxidised wear products. In sum, the NiCrMoNbTa coating present superior ambient dry sliding performance than the MCrAlY one.

### 3.3. Cavitation Erosion (CE) Resistance

**Figure 12:**
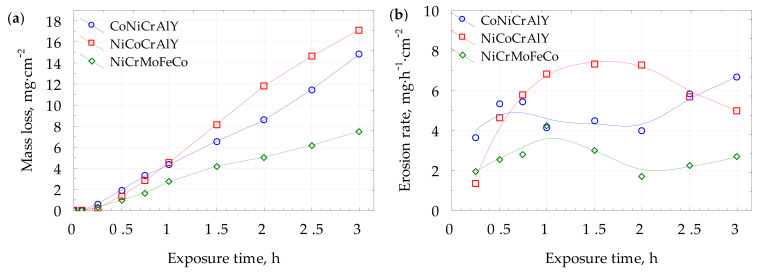
Cavitation erosion curves: (**a**) mass loss; (**b**) erosion rate plots.

**Figure 13:**
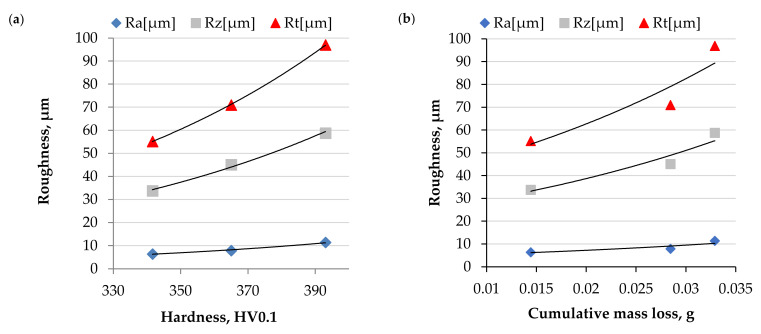
Effect of hardness (**a**) and material loss (**b**) on eroded surface roughness, 3 h of exposure.

The cavitation erosion (CE) results, presented in Figure 12a, indicate an almost twice lower material loss of NiCrMoNbTa than for the MCrAlY coatings. All investigated materials present negligible incubation time (Figure 12b), following the results obtained for plastic and ceramics coatings [45,50]. The cavitation damage varies in the erosion rates. The highest erosion rates were obtained for NiCoCrAlY and CoNiCrAlY, and the lowest for NiCrMoNbTa coating. The literature reports that in the case of similar types of materials, higher hardness facilitates cavitation erosion resistance. Additionally, research papers [30,66,67] report that in the case of different materials systems, hardness alone is not a direct indicator of cavitation erosion performance. The microstructure of investigated coatings’ is not similar and affects the hardness. Figure 13 confirms that the mass loss correlates with the decreasing mean hardness and the increase of the surface roughness of the eroded area. The highest material loss was obtained for the hardest coating (mean hardness of 393 HV0.1). The softest NiCrMoNbTa (mean hardness of 342 HV0.1) presents the lowest mass loss. As discussed in previous sections, the analysis of hardness variability indicates overlapping of standard deviations (see Figure 7), even though it seems that overall hardness can be employed as an indicator of material deformability. Moreover, the previous study regarding NiCrSiB alloys [32] confirms it. Softer nickel-based phases were prone to plastic deformation, which effectively mitigated cavitation damage. Overall, in the case of investigated HVOF metallic coatings containing nickel, hardness and deformability are essential factors for CE resistance. However, these factors derive from the microstructure of coatings, and microstructure features are responsible for cavitation erosion behavior.

**Figure 14:**
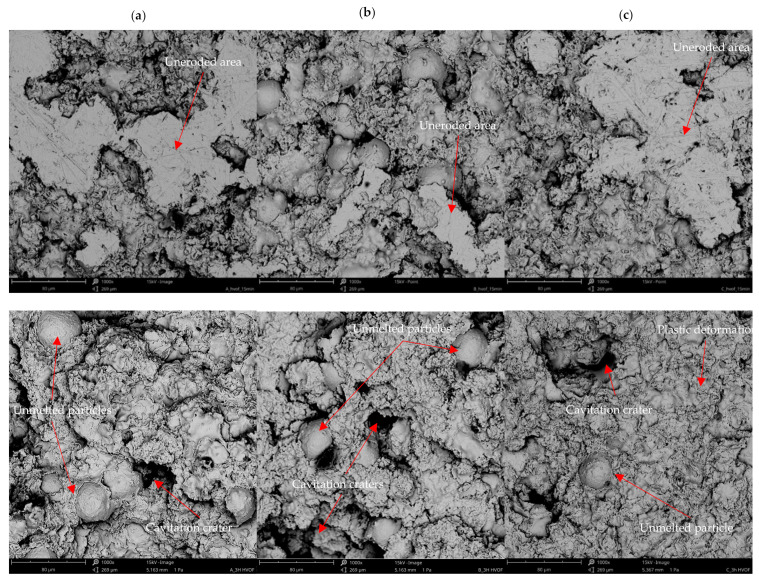

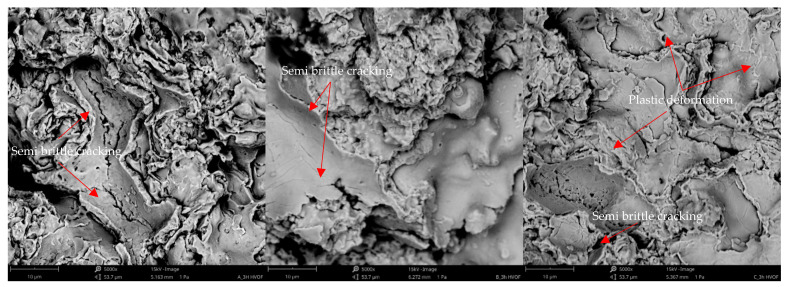
Comparison of eroded surfaces: (**a**) CoNiCrAlY, (**b**) NiCoCrAlY, (**c**) NiCrMoNbTa observed at 15 min and 180 min of cavitation erosion testing.

The erosion mechanism was analysed by combining SEM microscopic observations (Figure 14) with coating properties and profilometric measurements (Figure 13). Therefore, the presence of microstructure discontinuities, such as unmelted particles and pores results in the creation of deep pits and facilitates material removal, especially at the initial stages of erosion, see Figure 12b and Figure 14. At greater exposure times, severe pitting is observed. On the contrary, the well-melted splats undergo plastic deformation, which slows down the erosion rate (see Figure 14c). Finally, brittle cracking is visible in the NiCoCrAlY coating, which leads to material detachment. Profilometric measurements of eroded surfaces (Figure 13) confirmed that the NiCrMoNbTa coating was characterised by more uniform surface roughness and lesser pitting. Its plastic deformation slowed down the large craters’ growth and material loss. Consequently, a relatively soft NiCrMoNbTa coating shows an ability to consume the cavitation loads for plastic deformation (visible in Figure 14), while other coatings present much more brittle behaviour resulting in accelerating material detachment.

The severe erosion of the coatings relies on the formation of deep pits, and the coatings subject to more significant erosive damage were characterised by higher roughness parameters (see Figure 13). High nickel content facilitates the deformability of NiCrMoNbTa coating, resulting in ductile erosive behaviour. Contrary to this, MCrAlYs are characterised by lower deformability (higher mean hardness). Therefore, their failure mechanisms have a semi brittle mode which speeds erosion damage. It seems that microstructure features affect mechanical properties and determine the CE behaviour. Uniform microstructure and high deformability are crucial factors for increasing the CE resistance of thermally sprayed metallic coatings.

## 4. Conclusions

The thermally sprayed coatings, namely CoNiCrAlY, NiCoCrAlY, and NiCrMoNbTa, were comparatively examined in terms of the effect of microstructure and hardness on sliding wear and cavitation erosion resistance. As a result, the following conclusions can be drawn:

− The coatings present increasing resistance to both wear and cavitation erosion in the following order: NiCoCrAlY < CoNiCrAlY < NiCrMoNbTa.
− The NiCrMoNbTa and CoNiCrAlY coatings’ microstructure is dominated by a single-phase matrix and presents mean hardness of 342 HV0.1 and 365 HV0.1, respectively. Even though the NiCoCrAlY coating presents the highest mean hardness (393 HV0.1), it shows the worst tribological and cavitation erosion performance. The differences in microstructure of investigated coatings affect the wear and cavitation erosion performance more than the hardness itself.
− Superior sliding wear behaviour, i.e., lowest coefficient of friction and wear factor K, was noted for NiCrMoNbTa coatings. This coating presents abrasive grooving wear, adhesive smearing, and fatigue of oxidised wear products, while the NiCoCrAlY wear mechanism relies mainly on the abrasive grooving and oxidation of wear products which results in the highest wear rate and COF.
− The coatings’ microstructure affects mechanical properties and determines the CE behaviour. Cavitation erosion is initiated at microstructure discontinuities and ends up with severe surface pitting. MCrAlY coatings present semi brittle behavior, while NiCrMoNbTa coating shows ductile mode and lesser surface pitting.
− In the case of cavitation erosion, the hardness correlates well with the erosion results, i.e., softer coatings present higher deformability and display better performance under cavitation load by absorbing it for plastic deformation. Thus, NiCrMoNbTa coatings present the lowest cavitation erosion rate.

## Figures and Tables

**Table 1:** Nominal chemical composition of Inconel 617.

	Ni	Cr	Mo	Cu	Co	C	Mn	Si	S	Fe	Ti	Al	B
Wt.%	44.5	20–24	8–10	0–0.5	10–15	0.05–0.15	0–1	0–1	0–0.015	0–3	0–0.6	0.8–1.5	0–0.006

**Table 2:** Nominal chemical composition of feedstock powders used for HVOF spraying.

CoNiCrAlY		NiCoCrAlY		NiCrMoNbTa	
Element	(wt.%)	Element	(wt.%)	Element	(wt.%)
Ni	29.00–35.00	Ni	Bal.	Ni	Bal.
Co	Bal.	Co	22.00	Co	-
Cr	18.00–24.00	Cr	17.00	Cr	21.50
Al	5.00–11.00	Al	12.50	Al	-
Y	0.10–0.80	Y	0.55	Y	-
Mo	-	Mo	-	Mo	9.00
Nb + Ta	-	Nb + Ta	-	Nb + Ta	3.70
Fe	-	Fe	-	Fe	2.50

**Table 3:** Chemical composition of coatings estimated in spots marked in Figure 5, SEM-EDS.

CoNiCrAlY		NiCoCrAlY		NiCrMoNbTa	
Element	(wt.%)	Element	(wt.%)	Element	(wt.%)
Ni	35.15	Ni	47.49	Ni	64.56
Co	37.33	Co	22.14	Co	-
Cr	19.83	Cr	20.03	Cr	18.71
Al	7.68	Al	10.34	Mo	9.34
Y	-	Y	-	Nb	4.32
				Ta	2.81
				Fe	0.25

## Data Availability

Data is contained within the article.
